# Functional MRI of Letter Cancellation Task Performance in Older Adults

**DOI:** 10.3389/fnhum.2019.00097

**Published:** 2019-04-16

**Authors:** Ivy D. Deng, Luke Chung, Natasha Talwar, Fred Tam, Nathan W. Churchill, Tom A. Schweizer, Simon J. Graham

**Affiliations:** ^1^Physical Sciences Platform, Sunnybrook Research Institute (SRI), Toronto, ON, Canada; ^2^Department of Medical Biophysics, University of Toronto, Toronto, ON, Canada; ^3^Neuroscience Research Program, Keenan Research Centre for Biomedical Science, Toronto, ON, Canada; ^4^Institute of Medical Science, University of Toronto, Toronto, ON, Canada; ^5^Division of Neurosurgery, St. Michael’s Hospital, Toronto, ON, Canada; ^6^Institute of Biomaterials and Biomedical Engineering, University of Toronto, Toronto, ON, Canada

**Keywords:** letter cancellation, fMRI, brain mapping, neuropsychological tests, healthy aging

## Abstract

The Letter Cancellation Task (LCT) is a widely used pen-and-paper probe of attention in clinical and research settings. Despite its popularity, the neural correlates of the task are not well understood. The present study uses functional magnetic resonance imaging (fMRI) and specialized tablet technology to identify the neural correlates of the LCT in 32 healthy older adults between 50–85 years of age, and further investigates the effect of healthy aging on performance. Subjects performed the LCT in its standard pen-and-paper administration and with the tablet during fMRI. Performance on the tablet was significantly slower than on pen-and-paper, with both response modes showing slower performance as a function of age. Across all ages, bilateral brain activation was observed in the cerebellum, superior temporal lobe, precentral gyrus, frontal gyrus, and occipital and parietal areas. Increasing age correlated with reduced brain activity in the supplementary motor area, middle occipital gyrus, medial and inferior frontal gyrus, cerebellum and putamen. Better LCT performance was correlated with increased activity in the middle frontal gyrus, and reduced activity in the cerebellum. The brain regions activated are associated with visuospatial attention and motor control, and are consistent with the neural correlates of LCT performance previously identified in lesion studies.

## Introduction

Cancellation tasks have long been used in psychology research, as complex probes of attention that combine both visual selectivity and motor response. Administered in paper-and-pencil format, these tasks consist of patterns of letters, numbers or symbols interspersed with a target letter, number or symbol, with the instruction to cross out (cancel) all of the targets. Task difficulty can be changed by varying the spacing and frequency of the targets; for example, if the targets are made more sparse, then the task becomes more difficult. Task performance can be scored straightforwardly by using measures such as the completion time and the number of errors. Lower scores can reflect various underlying factors such as general response slowing and inattentiveness from diffuse damage or acute brain conditions, or specific deficits in response shifting, motor smoothness, or unilateral attention (Lezak, 1995).

The Letter Cancellation Task (LCT) concept was first described and implemented by Talland (1965). Shortly thereafter, Diller et al. (1974) constructed nine different cancellation tasks (using letters, numbers, words, shapes and pictures), including the single letter LCT that is presently in wide use. The basic task consists of six 52-letter rows, with the target letter randomly interspersed 18 times in each row. Other cancellation tasks present items in random spatial distributions rather than in linear rows, such as the symbol cancellation test developed by Mesulam (1985).

As for all neuropsychological assessment tools, the neural correlates of LCT performance are of interest to inform clinicians about the areas of the brain that are potentially affected in patients. For example, the neural correlates of the LCT have been discussed historically in the context of patients with stroke. Such studies suggest that right hemisphere lesions result in a greater number of errors on the LCT due to spatial neglect, while left hemisphere lesions result in a longer time to completion, associated with temporal processing deficiencies (Diller et al., 1974). However, there is still no consensus on the specific neural correlates of LCT errors arising from neglect. Most lesion studies of left neglect implicate the right inferior parietal lobe or angular gyrus (Mort et al., 2003; Molenberghs and Sale, 2011), and right temporoparietal junction or superior temporal gyrus (Karnath et al., 2001; Rousseaux et al., 2015). Other studies implicate frontoparietal tracts which connect the inferior parietal lobule, associated with spatial attention, with the premotor and prefrontal cortices, associated with spatial working memory (Corbetta and Shulman, 2011; Urbanski et al., 2011).

There are fundamental weaknesses in relying on lesion studies to identify the neural correlates of neuropsychological test performance. Cases have been reported where lesions in different locations present similar test results, and others where different results are obtained for lesions in similar locations (Lezak, 1995). The lesions in patients are not experimentally controlled (compared to what is achievable in animal models) and vary in location and extent, rarely conforming to functionally homogenous neuroanatomical regions. In addition, patients with lesions often have more than one functional deficit and may employ compensatory mechanisms to maintain behavior. It is necessary, therefore, to seek other scientific evidence to corroborate and interpret the neural correlate results derived from lesion studies.

One important strategy toward this goal involves using functional neuroimaging methods to assess the relationship between brain structures and LCT performance. However, although performance on the LCT has been correlated with functional magnetic resonance imaging (fMRI) activation maps of stroke patients, implicating the right superior parietal cortex (Hassa et al., 2011), no studies have mapped and reported the brain activity that occurs directly while the LCT is being performed. In part, this gap in knowledge may have arisen due to the lack of appropriate peripheral device technology, and the challenges in performing paper-and-pencil behavioral testing during fMRI. Specialized devices that support realistic writing and drawing behavior have been developed in recent years, however, enabling test performance during fMRI that is similar to standard office testing conditions. The device of interest here is an fMRI-compatible tablet with a touch-sensitive screen and stylus, accompanied by a video camera for augmented reality display that provides visual feedback of hand and stylus position during tablet interactions (Tam et al., 2011; Karimpoor et al., 2015). This tablet system has been validated and used in multiple fMRI studies, for example to evaluate the neural correlates of the Trail Making Test, also widely used in neuropsychology (Karimpoor et al., 2017, 2018).

Previous studies of LCT performance in healthy adults have shown an effect of age on performance, such that older adults exhibit longer completion times (Geldmacher and Riedel, 1999; Uttl and Pilkenton-Taylor, 2001). These results are consistent with other findings of age-related declines in speed and efficiency for visual-spatial search tasks (Plude and Hoyer, 1986; Madden, 1990). Whereas electroencephalography (EEG) studies have been conducted to identify neural correlates of visual attention declines in older adults (Wiegand et al., 2014; Hong et al., 2015), little is known about the mechanisms of slowing that are specific to the LCT. With the added benefit that fMRI provides maps of brain activity at higher spatial resolution than EEG, it is of interest to explore the neural correlates of LCT performance in relation to healthy aging. Such a pursuit also provides baseline normative data suitable for comparison with results obtained from patients suffering from brain diseases prevalent in the elderly, such as stroke.

The present work thus characterizes the neural correlates of LCT performance during fMRI using the above-mentioned tablet system, in a cohort of healthy elderly individuals. It is hypothesized that: (a) tablet-based LCT performance during fMRI will correlate with standard LCT performance outside of the magnet; (b) areas activated during LCT performance in fMRI will be consistent with previous lesion studies, and fMRI studies of behavioral components elicited by the LCT; and (c) brain activity will show changes across the cohort consistent with age-related slowing in LCT performance.

## Materials and Methods

### Subjects

Thirty-two healthy volunteers (15 males, 17 females; mean age ± standard deviation 73.1 ± 9.3 years) participated in this fMRI study, which was approved by the Research Ethics Board at St. Michael’s Hospital in Toronto. All subjects met standard MRI exclusion criteria (e.g., free from claustrophobia, ferromagnetic implants). Additional exclusion criteria included: (1) history of a severe neurological incident (e.g., traumatic brain injury, stroke, brain tumor); (2) history of any neurological condition (e.g., Alzheimer’s disease, Parkinson’s disease, multiple sclerosis); (3) history of any serious psychiatric condition (e.g., bipolar disorder, schizophrenia); (4) presence of a gross motor disorder; (5) history of substance abuse or dependence; (6) any visual abnormalities not corrected with lenses; and (7) any significant hearing loss. Subjects were recruited from the Baycrest Geriatric Hospital Research Volunteer Database, the St Michael’s Hospital Volunteer Association, the University of Toronto Alumni Association, and an advertisement in Zoomer magazine under the Canadian Association for Retired Persons (CARP). All subjects were fluent in English and were right-handed based on administration of the Edinburgh Handedness Inventory (Oldfield, 1971). The Montreal Cognitive Assessment (MoCA) was also administered to assess cognitive status (Nasreddine et al., 2005). Three subjects (1 female, 2 males) were excluded from further analysis because of their low MoCA scores compared to normative data, or due to failure to complete the study.

### Experimental Task

Prior to the fMRI session, subjects performed the LCT with pen-and-paper in a psychometric testing office. Subjects were instructed to cross out the single letter “H,” which was presented 104 times among an array containing six lines of 52 letters. The completion time was recorded. Subjects were then briefly trained on the tablet outside of the magnet for 30 s with a similar version of the LCT involving the target “I.”

**Figure 1:**
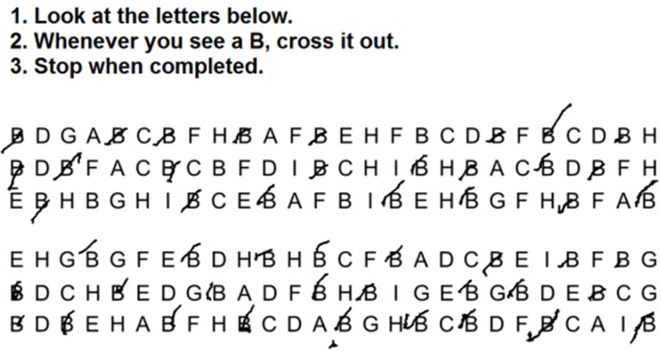
Sample tablet-based Letter Cancellation Task (LCT) stimuli and responses from a single representative subject performing the experimental task during functional magnetic resonance imaging (fMRI), in this case with instructions to cancel out all instances of the letter “B.”

Additional training was performed using the tablet inside the magnet, including various writing and drawing tasks (writing your name, crossing out shapes, connecting dots, tracing a flower, and drawing a house). Subjects then performed the tablet LCT during fMRI for different arrays of letters in five separate runs, each involving a different target letter (see Figure 1). Each row of the array contained nine target letters, evenly spaced, with similar letters around it. This experimental task had a fixed block duration of 60 s per array. Each LCT task was separated by 20-s periods of visual fixation on a centered cross. The experiment was implemented using a custom program written with E-prime Software (version 2; Psychology Software Tools, Inc., Sharpsburg, PA, USA). After the fMRI session, 16 of the subjects completed a post-experiment questionnaire which evaluated their experience using the tablet, and their self-rated performance on the tablet-based task compared to the paper task.

### fMRI-Compatible Tablet Technology

The fMRI-compatible tablet system previously developed in the laboratory consists of a touch-sensitive surface, stylus and an MRI-compatible video camera for visual feedback of hand position (Tam et al., 2011; see Karimpoor et al., 2015; for complete set-up details). The touch-sensitive surface converts the force of contact into position coordinates that can then be located on a computer display for subsequent drawing on the screen. The video camera is equipped with a color CMOS sensor (12M-i, MRC Instruments Gmbh, Germany) and a custom LED illuminator. The video signals are sent through a color detection algorithm to isolate the hand and stylus, which are then graphically superimposed on the visual stimuli, for real-time presentation to the subject in augmented reality. The visual stimulus presentation was achieved using an fMRI-compatible projection system (Avotec, Stuart, FL, USA), an angled mirror mounted on the head coil receiver of the MRI system, and a rear display screen providing a visual angle of 15.5°.

### fMRI Parameters

All imaging was performed at St. Michael’s Hospital using a 3.0 T MRI system (Magnetom Skyra, Siemens Healthineers, Erlangen, Germany) with the standard 20-channel head coil. A T1-weighted anatomical brain image was first acquired using a three dimensional magnetization-prepared rapid gradient echo protocol [inversion time (T1) = 1,090 ms, echo time (TE) = 3.55 ms, repetition time (TR) = 2,300 ms, flip angle (FA) = 80°, bandwidth (BW) = 200 Hz/pixel, field of view (FOV) = 240 × 240 × 173 mm, acquisition matrix = 256 × 256 × 192, isotropic voxel dimension = 0.9 mm], providing an underlay for the subsequent fMRI maps of brain activity. The fMRI time series data were then acquired using multi-slice T2*-weighted Echo-Planar Imaging (EPI; TE = 30 ms, TR = 2,000 ms, flip angle (FA) = 70°, bandwidth (BW) = 2298 Hz/pixel, oblique-axial orientation, 32 interleaved ascending slices with FOV = 200 × 200 mm, acquisition matrix = 64 × 64, slice thickness = 4.0 mm with 0.5 mm gap, 3.125 mm × 3.125 mm × 4.5 mm voxel size). For additional physiological monitoring during fMRI, each subject also had a photoplethysmograph attached to a finger on the left hand and a respiratory cushion strapped to the torso.

### Behavioral Data Analysis

Both the paper and tablet versions of the LCT were scored by counting the number of hits (canceled target letters), omissions (target letters not canceled) and commissions (non-target letters canceled). The tablet version of the LCT was scored by two different trained test administrators with a high inter-rater reliability (Pearson’s *r* = 0.92 for the number of hits) and scores were averaged across the two raters. Because not all subjects completed all tablet LCT arrays within each 60-s block duration, an additional metric was developed to compare performance on the paper and tablet versions of the LCT. The “seconds per hit” (sph) value was computed over the appropriate time interval (the completion time for the paper version of the LCT, and five block durations (300 s) for the tablet version, divided by the number of hits achieved). A paired two-sample *t*-test was then performed to investigate whether there were statistically significant differences between sph values. Pearson’s bivariate correlation was also computed to investigate whether there was consistency in performance of the paper and tablet versions of the LCT across subjects.

In addition, a repeated-measures analysis of covariance (ANCOVA) was performed assess the effect of LCT version and age on sph values, along with potential interaction effects between these factors. All statistical testing was performed using RStudio (RStudio, Inc., Boston, MA, USA), an open-source computing environment for the statistical software R (R Core Team, 2008).

### fMRI Data Analysis

The fMRI data and structural scans were manually inspected for any visual abnormalities. With none found, data preprocessing and analysis were then performed using a hybrid pipeline which includes tools from the Analysis of Functional Neuroimages (AFNI^1^) package (Cox, 1996), the FMRIB Software Library (FSL^2^) package (Smith et al., 2004), and algorithms custom-written in the laboratory. The preprocessing pipeline incorporated slice-timing correction (AFNI 3dTshift), rigid-body motion correction (AFNI 3dvolreg), spatial smoothing (AFNI 3dmerge), removal of outlier scan volumes *via* SPIKECOR^3^ and regression of motion parameters and linear-quadratic trends as nuisance covariates. To control for physiological noise: (a) PHYCAA+^4^ was used to perform data-driven down-weighting of regions other than gray matter (Churchill and Strother, 2013); and (b) seed analysis was incorporated into the pre-processing using regions of interest in the left and right corona radiata and the left and right lateral ventricles. The resulting data were transformed into a common anatomical template space as follows: the FSL flirt algorithm was used to calculate the rigid-body transform of the mean fMRI volume to the T1-weighted anatomical image, and the affine warp of the T1 anatomical image to the MNI152 Montreal Neurological Institute (MNI) template for each subject (Mazziotta et al., 2001). The net transformation matrices were applied to the fMRI data, which were resampled at a spatial resolution of 3 × 3 × 3 mm. Due to the variability in brain size among subjects >80 years old, the anatomical transformation was improved by manual inspection and manual segmentation of the brains, if required.

Analysis of the preprocessed imaging data at the individual subject level (first level) was completed by fitting the task conditions (LCT and fixation) in a general linear model (GLM) to achieve the contrast of LCT performance vs. fixation. The time series data were edited so that individual activation maps were generated only for the time duration in each task block that each subject was engaged in LCT performance, which ranged between 33 s and 60 s. Time series data that were collected after task completion during the task block were not included in the analysis. The analysis was done using the OPPNI (Churchill et al., 2012a,b, 2015), and NPAIRS analysis framework (Strother et al., 2002), which estimated coefficient maps from split-halves of the fMRI time series data and used these maps to compute reproducible, z-scored maps of reliable task-related activation for each subject. Group-level (second level) analysis was then done by performing 1-sample bootstrap analyses on the z-scored subject maps for the whole group. Bootstrapping provides a non-parametric estimate of group-level effects, which avoids data distributional assumptions. This is done by resampling on subjects with replacement (1,000 iterations) to obtain an empirical *p*-value. Significant voxels were then identified by first applying a voxel-wise threshold of *p* < 0.005, followed by cluster size thresholding at a minimum cluster size of 1,764 mm^3^ to control for multiple comparisons at a family-wise *p* < 0.05 threshold (obtained by estimating spatial smoothness of the data using AFNI 3dFWHMx, followed by determination of minimum cluster size using AFNI 3dClustSim). For significant voxels, the bootstrap ratios (BSRs) were reported (calculated as the bootstrap mean divided by standard error), providing a normally-distributed standardized measure of effect size.

Group-level covariate analysis was also performed by conducting voxel-wise ordinary least squares linear regression of both age and tablet-based LCT performance scores (sph values) against individual subject activation z-scores, within a region of interest (ROI) mask created from the clusters identified as significant in the 1-sample group analysis. For this analysis, significance was also evaluated for each regressor by performing non-parametric bootstrap analysis on the regression coefficients to obtain empirical *p*-values, with cluster-size thresholding as described above, and BSRs were reported for voxels identified as significant.

## Results

### LCT Performance

**Figure 2:**
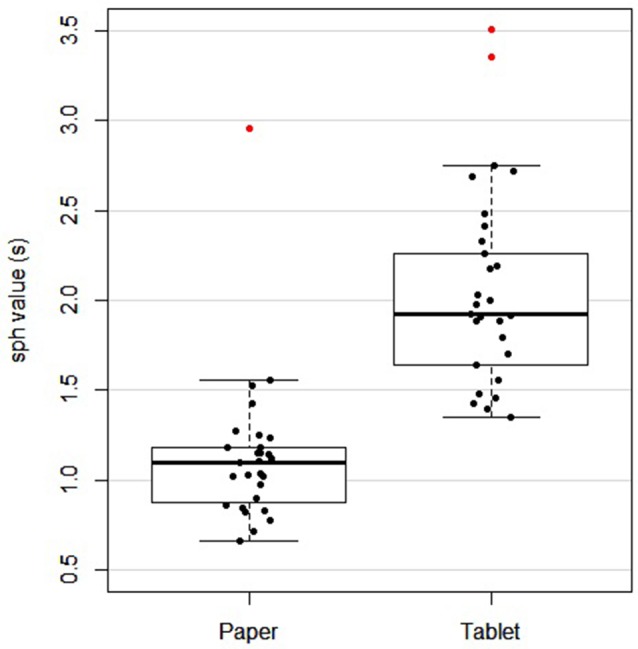
Box and whisker plot, and scatter plot of subject performance [seconds per hit (sph) value] for paper and tablet versions of the LCT. For each box, the interior line in bold shows the median, and the edges of the box are estimates of the first and third quartiles. The whiskers extend to the most extreme data points not considered outliers (1.5 times the interquartile range from the edges of the box). One subject was excluded as they did not have a paper LCT score. Outliers are shown in red.

Table 1 lists the demographic data and neuropsychological assessment scores of all the subjects. Overall, subjects performed the paper version of the LCT in a median completion time of 113.9 s with a no omissions and no commissions. The tablet version was performed with a median of 3.2 omissions and 1.1 commissions, over all subjects and the five task blocks included in the fMRI experiment. Because the tablet version of the LCT had a fixed task block duration of 60 s, and involved the five task blocks (whereas the paper version required all subjects to perform the task once, to completion), quantitative comparison of omission and commission errors was not attempted between the versions. Instead, the sph values were used to provide a direct comparison. The median and IQR sph values for the paper and tablet versions were 1.1 (0.3) s and 1.9 (0.7) s, respectively. One subject did not complete the paper version and was excluded from further analysis of LCT performance comparing the two versions. Figure 2 shows a scatter plot of these data for each subject, as well as for the group in box-and-whisker format. Although there is some overlap in the sph distributions for each LCT version, a difference was observed based on the paired *t*-test (*t*_(27)_ = −7.37, *p* < 0.001), indicative of slower performance when using the tablet. When Pearson’s bivariate correlation was computed between sph values for the paper and tablet LCT versions for each subject, no significant effect was observed (*r* = 0.06, *p* = 0.745).

**Table 1:** Demographic data for the subjects included in the study.

	Median (IQR)	Quartile 1	Quartile 3
Age	76 (14.0)	66.0	80.0
Sex (female), *n* (%)	16 (55.2%)		
Years of education	16.0 (4.0)	13.0	17.0
MoCA score	27.0 (2.0)	26.0	28.0
**Paper LCT**			
Completion Time (s)	113.9 (30.9)	93.1	124.1
Omissions	0 (0)	0	0
Commissions	0 (0)	0	0
Seconds per hit	1.1 (0.3)	0.9	1.2
**Tablet LCT**			
Omissions	3.2 (2.9)	1.4	4.3
Commissions	1.1 (2.7)	0.6	3.3
Seconds per hit	1.9 (0.7)	1.6	2.3

*Values reported in median (interquartile range, IQR) format unless otherwise stated. n, number of observations; MoCA, Montreal Cognitive Assessment; LCT, Letter Cancellation Task*.

**Figure 3:**
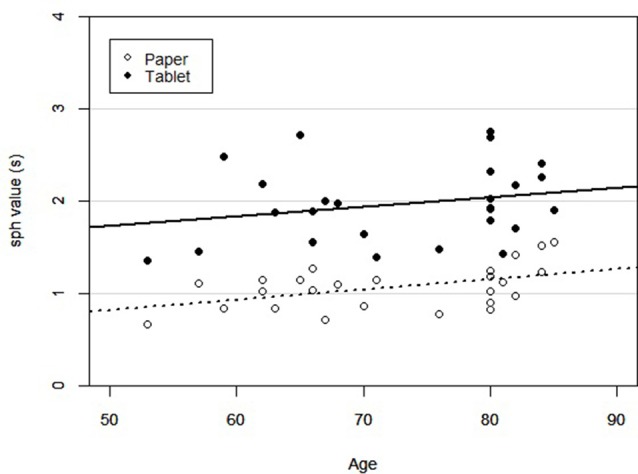
Scatter plot of subject LCT performance across age for each version of the LCT (paper and tablet). Linear regression lines were statistically significant in both cases (*p* < 0.05).

Figure 3 shows regression plots of sph values for both LCT versions vs. age, supporting the ANCOVA that was conducted. Outliers determined from Figure 2 were excluded from the ANCOVA. There was an effect of LCT version on performance after controlling for age (*F*_(1,23)_ = 112.30, *p* < 0.001), with slower performance on the tablet version compared to the paper version. There was also an effect of age on LCT performance, with performance slowing (increasing sph values) with greater age (*F*_(1,23)_ = 4.87, *p* < 0.05). *Post hoc* linear regressions of sph vs. age were also statistically significant for both versions (*p* < 0.05). For the paper version, the estimated slope and intercept were 0.0103 ± 0.0043 s/year and 0.3130 ± 0.3031 s, respectively, with the experimental uncertainty given by the standard error. For the tablet version, the analogous values were 0.0101 ± 0.0085 s/year and 1.2295 ± 0.6303 s. The interaction effect between age and LCT version was not significant (*F*_(1,23)_ = 0.011, *p* = 0.918).

Overall, subjects self-reported that their performance on the tablet-based LCT was good, and not affected by the switch from the paper to the tablet (Table 2). Subjects also reported that the tablet set-up was both comfortable and usable, and that the tablet session did not cause any adverse physiological symptoms.

**Table 2:** Post-experiment questionnaire results, completed by 16 subjects from the study.

	Median (IQR)	Quartile 1	Quartile 3
**Quality of tablet-based LCT performance**	4 (1)	4	5
1 = very poor			
2 = poor			
3 = fair			
4 = good			
5 = excellent			
**Quality of LCT performance on the tablet compared to on paper**	3 (0)	3	3
1 = much worse			
2 = slight worse			
3 = the same			
4 = better			
5= much better			
**Experimental Set-Up**			
1 = very poor			
2 = poor			
3 = fair			
4 = good			
5 = excellent			
Quality of visual display	4 (1)	3	4
Comfort with position of tablet	4 (0.5)	3.5	4
Comfort with using stylus	3 (1)	3	4
**Physiological Experience**			
1 = none			
2 = mild			
3 = moderate			
4 = severe			
Headache	1 (0)	1	1
Eyestrain	1 (1)	1	2
Difficulty focusing (visual)	2 (1)	1	2
Difficulty focusing (mental)	1 (1)	1	2
Dizziness	1 (0)	1	1
Fatigue after completion of the MRI session	2 (1)	2	3

*Values reported in median (interquartile range, IQR) format unless otherwise stated. LCT, Letter Cancellation Test; MRI, magnetic resonance imaging*.

### Brain Activity

**Figure 4:**
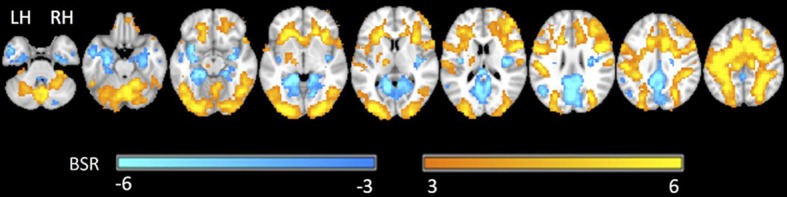
Representative statistical parametric maps of the group LCT vs. fixation contrast. Neurological convention is employed in Montreal Neurological Institute (MNI) coordinates. The bootstrap ratio (BSR) color bar is shown at the bottom. LH, left hemisphere; RH, right hemisphere.

Figure 4 shows the group statistical maps of brain activity (BSR values for LCT contrasted with visual fixation) for selected slice locations. The positive brain activity was found to be spatially extensive and predominantly bilateral, in areas including the precentral gyrus, postcentral gyrus, cerebellum, superior temporal lobe, frontal gyrus, and various occipital and parietal areas. Negative activation was also predominantly bilateral, involving the angular gyrus, thalamus, cerebellum, inferior frontal gyrus and areas of the temporal lobe.

**Figure 5:**
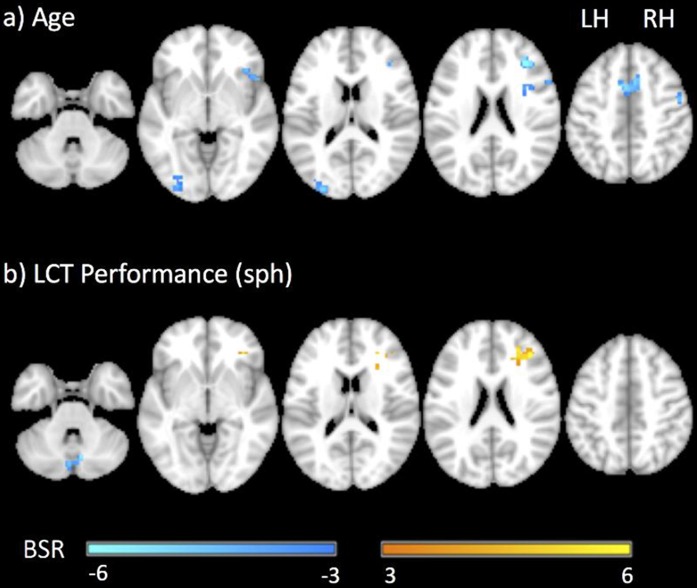
Representative statistical parametric maps of the LCT vs. fixation contrast showing the covariance of brain activity with **(A)** age and **(B)** LCT performance (sph value). Neurological convention is employed in MNI coordinates. The BSR color bar is shown at the bottom. LH, left hemisphere; RH, right hemisphere.

Figure 5 shows the group brain map results from the GLM covariate analysis, depicting the areas in which activation signal changed with: (a) age; and (b) LCT performance (sph value). The areas that showed an effect of age included the right middle and inferior frontal gyri, supplementary motor area, left middle occipital gyrus, cerebellum and putamen. All the areas that were identified showed an age-related reduction in brain activity. There were fewer areas that showed an effect of LCT performance, including increased activity in the right middle frontal gyrus, and reduced cerebellar activity. Table 3 reports all clusters of activation that were significant in the covariate analysis, together with each spatial location, cluster size and peak BSR value.

**Table 3:** Active brain regions identified for the contrast of LCT vs. fixation with age and LCT performance (sph value) as covariates. Value and location of the peak bootstrap ratio (BSR) of each cluster are reported in MNI space.

Active region	Hemisphere	Cluster size (mm^3^)	Peak MNI coordinates			BSR
			*x*	*y*	*z*	
**Age**				
Supplementary motor area	R	6,021	6	9	51	−6.52
Middle occipital gyrus	L	5,616	−30	−90	3	−6.06
Middle frontal gyrus	R	4,347	33	36	24	−7.69
Cerebellum VIII	L	2,727	−12	−66	−45	−6.57
Inferior frontal gyrus, pars opercularis	R	2,349	57	15	30	−8.85
Middle frontal gyrus	R	1,863	42	0	57	−7.12
Putamen	R	1,647	27	18	6	−5.86
**LCT performance**				
Middle frontal gyrus	R	2,808	30	33	18	5.62
Cerebellum VIIB	L	1,647	−6	−81	−45	−7.26

*R, right; L, left*.

## Discussion

This is the first fMRI study that directly characterizes the brain activity during LCT performance, using tablet technology to simulate standard pen and paper administration. The behavioral findings are considered in the discussion immediately below, followed by an appraisal of the fMRI results in relation to previous work investigating the neural correlates of the LCT.

### LCT Performance

The behavioral results found for healthy elderly individuals performing the standard paper version of the LCT (Table 1) are in agreement with published norms (Uttl and Pilkenton-Taylor, 2001). The expectation was to replicate these results with the tablet; however, the subjects took somewhat more time to strike out letters on a “per hit” basis, and with greater inter-subject variability when the tablet was used. Several factors may have contributed to these findings. For example, performance may have been affected by the need to limit the usable touch-sensitive surface area of the tablet (120 cm^2^) within the confined magnet bore while still showing all the task stimuli, increasing the demand for precise movements (Karimpoor et al., 2017). The mode of tablet interaction (in an augmented reality environment with visual feedback of hand position) has been shown to facilitate placement of the stylus tip when making responses (Karimpoor et al., 2015) but also can cause some of the array of letters to be blocked by the hand and forearm of the subject as the task is being performed. This was previously identified as a contributing factor to slower-than-expected performance in a recent study investigating tablet-based fMRI of the Trail Making Test, a test that also includes a visual search component (Karimpoor et al., 2017). Given the different viewpoints from which subjects performed the LCT in the paper and tablet versions (obliquely vs. top-down, respectively), subjects may have used slightly different strategies in the two cases to scan the array of letters for targets. Tablet LCT performance may also have been affected by the visual acuity of subjects in the magnet bore, even though fMRI-compatible lenses were provided to subjects to correct their vision. However, no subjects reported any major difficulties in performing the task in the post-experiment questionnaire.

Furthermore, no significant correlation between the tablet LCT and paper LCT performance was observed across subjects. A significant correlation was expected but may have been obscured by the small sample size (*n* = 29) in relation to the variability in the tablet data. On closer inspection, substantial variations in tablet performance were observed across the five blocks in the fMRI design. On average, the standard deviation of the sph value was 49% of the mean per subject, with no obvious practice effects or systematic effects observed. It is not possible to assess whether these observations are in agreement with repeated performance on the standard version of the LCT, as to the best of our knowledge there have been no studies published of LCT test-retest reliability. Previous studies have instead investigated the convergent validity of the LCT with other neuropsychological tests (Uttl and Pilkenton-Taylor, 2001). A study of practice effects and test-retest reliability of the LCT would be useful in the future.

Nevertheless, the behavioral performance results did reveal a significant, consistent effect of age as a covariate for both standard paper and tablet versions of the LCT. The observed effect of slower task performance with increasing age is in agreement with literature on age-related slowing including slower or less efficient visual search (Geldmacher and Riedel, 1999), and general decline in attention, executive function, processing speed and visuospatial processing as a result of healthy aging (Harada et al., 2013).

These collective findings indicate that the tablet system provides a reasonable method for studying drawing behavior of the type required to perform the LCT during fMRI. The tablet provides performance that is reasonably similar to performance on the standard paper version of the LCT, and is similarly able to detect age-related slowing effects. However, the present tablet system enables behavior that is an approximation of (and not identical to) pen-and-paper performance conducted in a psychometric office testing environment, with some additional task demand and additional performance variability. The tablet-based fMRI data for LCT performance are subsequently interpreted with this proviso in mind.

### Brain Activity

The brain regions activated in the “all ages” group map of LCT vs. fixation contrast are generally consistent with expectations. As a test of attention, it is not surprising that the LCT activations overlap with the fronto-parietal attention network, including the supplementary motor area, prefrontal cortex, parietal lobule and temporal lobe (Li et al., 2015). The bilateral activations in the superior parietal lobule are associated with selective, goal-directed spatial attention and visuomotor control of eye-hand movements as a part of the dorsal fronto-parietal attention network (Corbetta and Shulman, 2011), which is particularly implicated in visual search tasks of attention (Posner and Petersen, 1990). Activations in the frontal cortex and temporo-parietal junction make up the ventral component of the network, directing attention to behaviorally relevant stimuli (Corbetta and Shulman, 2011). The lateral superior frontal areas and anterior cingulate have also been associated with tasks involving language and target detection (Posner and Petersen, 1990). The observed activation of the thalamus is also consistent with the literature, playing a role in coordinating the dorsal and ventral components of the attention network (Posner and Petersen, 1990; Kastner and Pinsk, 2004).

Previous lesion studies also provide support for the observed activations relating to visuo-spatial and visuo-motor requirements of the LCT. The activations of the inferior parietal lobule, superior temporal gyrus and middle occipital gyrus are consistent with lesion studies involving patients with spatial neglect (Mort et al., 2003; Molenberghs et al., 2012). Activations of the frontal gyrus, precentral gyrus and supplementary motor area are also consistent with studies of target cancellation tasks, which found that cancellation deficits arose preferentially from motor exploration problems associated with frontal lesions (Binder et al., 1992; Molenberghs et al., 2012).

However, past lesion studies that use the LCT to probe neglect have implicated the right hemisphere, whereas the present fMRI study found predominantly bilateral activations associated with LCT performance in the healthy elderly. The precise loci responsible for neglect are of some controversy, but bilateral activation is certainly expected for the fronto-parietal and occipital regions in healthy subjects. Although shifts of attention are managed by the hemisphere contralateral to the side of visual field, the LCT utilizes visual stimuli that are presented on both the left and right sides (Scolari et al., 2015). The bilateral activation in the sensorimotor areas (precentral gyrus, postcentral gyrus, cerebellum, supplementary motor area) is perhaps more surprising for a task that elicits unilateral motor responses. Yet previous fMRI studies have found that tasks with higher demand for precise motor responses do result in bilateral activations, with recruitment of ipsilateral motor areas for planning and execution (Horenstein et al., 2009; Buetefisch et al., 2014). The present data suggest that the effect also extends to the somatosensory system; this may be plausible given the intimate association between primary motor and primary somatosensory cortex, and that considerable proprioceptive and tactile input are required to make tablet responses precisely in space. Furthermore, somatosensory regions may also play a role in directing visuospatial attention, beyond the traditional role of processing sensory input (Balslev et al., 2013).

The group map of LCT vs. fixation also included multiple negative activations (greater activation during fixation than during LCT performance) in the inferior frontal gyrus, inferior temporal gyrus, thalamus, angular gyrus, cerebellum and hippocampus. The inferior frontal gyrus, anterior temporal lobe, angular gyrus and hippocampus are often activated during wakeful rest (Uddin et al., 2009; Xu et al., 2016) and are components of the default mode network (DMN). The DMN has been linked to processes involving memory (Greicius and Menon, 2004), future planning (Xu et al., 2016), reflection and task-unrelated thought (McKiernan et al., 2003). In contrast, the negative activation of the cerebellum is consistent with its role in ocular motor control to help hold the gaze for fixation (Kheradmand and Zee, 2011).

Turning now to the covariance analysis results, reductions in brain activity with increasing age were observed in the frontal gyrus, middle occipital gyrus, supplementary motor area and cerebellum. These results are expected, and consistent with the age-related decline in LCT performance that was observed in the behavioral data. The reduced activation in the right frontal gyrus corresponds to the fronto-parietal attentional network that supports LCT performance, similar to age-related declines in attention observed with other fMRI studies (Solbakk et al., 2008; Nyberg et al., 2010). The reduced activity in the supplementary motor area and cerebellum is also consistent with age-related declines in motor processing. The underlying mechanisms responsible for these effects in healthy aging—including volumetric changes in brain regions, reductions in functional connectivity, increases in inflammation and reductions in cerebral perfusion—are complex, extensively studied, and beyond the scope of the present study (Lindenberger, 2014). Notably, some of the physiological factors just mentioned may also impact the BOLD signal with less influence on the underlying neural activity (e.g., perfusion; Ances et al., 2009), and the lack of controls for such effects constitute a limitation of the present work. Additional research will be required in the future with appropriate physiological imaging controls to corroborate the study findings.

Independent of the covariance with age, multiple brain areas were observed to have activity that covaried with LCT performance, as quantified by sph values. There were two patterns of effect: (a) positive correlation of brain activity with sph value (decreased LCT performance), found in the right middle frontal gyrus; and (b) negative correlation of brain activity with sph value (increased LCT performance), found in the cerebellum. The increased activity in the right middle frontal gyrus with decreased LCT performance suggests a greater need to recruit the area when the task is found to be difficult. This is consistent with the results of a recent study employing transcranial direct current stimulation (tDCS), which showed that stimulation of the right prefrontal cortex caused impaired performance in tasks that were found to be cognitively demanding on an individual subject basis, independent of task type (Vergallito et al., 2018). Conversely, the decreased cerebellar activity is important as this region is critically implicated in the processing of highly skilled visuomotor tasks. It is reasonable to suggest that individuals with more visuomotor skill at performing the LCT therefore had more efficient mental processing in this area, and thus activated it to lesser extent than those individuals who performed the LCT more poorly.

It is also important to indicate that our interpretations of the study findings are speculative, given the initial nature of the experiments. Additional fMRI studies of the LCT will be required to replicate the present findings, and to refine understanding of the neural correlates of the test. As part of such future work, more detailed behavioral recording will be useful (e.g., including fMRI-compatible eye-tracking), together with the use of carefully designed control tasks (e.g., that closely match visual input, movement kinematics and stylus contact force to those of LCT performance), to study the interplay between visuomotor scanning and motor responses and the related brain activation dynamics. In addition, studies involving non-invasive neural stimulation (e.g., tDCS or TMS) will be useful to evaluate the functional roles of the activated regions found by fMRI.

## Conclusion

The present work is the first to identify neural correlates of the LCT using fMRI and tablet technology in a healthy aging population. Across all ages, the activation was found to be bilateral, including in the cerebellum, superior temporal lobe, precentral gyrus, frontal gyrus, and various occipital and parietal areas. With increasing age, performance generally decreased and brain activity was reduced in the supplementary motor area, middle and inferior frontal gyrus, middle occipital gyrus, putamen and cerebellum. Better LCT performance was correlated with increased activity in the middle frontal gyrus, and reduced activity in the cerebellum. Overall, these data provide important normative baseline information that will be useful in subsequent evaluations of abnormal brain and behavior relationships, when the LCT is administered to elderly patients.

## Ethics Statement

The study was approved by the Research Ethics Board at St. Michael’s Hospital in Toronto, Canada. All subjects gave written informed consent in accordance with the REB guidelines and the Declaration of Helsinki.

## Author Contributions

NT: data collection. NT, LC, ID and NC: analysis. NT, FT, NC, SG and TS: experimental design. ID, LC and SG: manuscript preparation. ID, SG, FT, NC and TS: evaluation and interpretation of results. All authors edited the manuscript.

## Conflict of Interest Statement

The authors declare that the research was conducted in the absence of any commercial or financial relationships that could be construed as a potential conflict of interest.

## Footnotes

^1^https://afni.nimh.nih.gov

^2^https://www.fmrib.ox.ac.uk/fsl

^3^https://www.nitrc.org/projects/spikecor_fmri

^4^https://www.nitrc.org/projects/phycaa_plus
